# *Corema album* Berry Juice as a Protective Agent Against Neurodegeneration

**DOI:** 10.3390/ph17111535

**Published:** 2024-11-15

**Authors:** Antonio Canoyra, Carmen Martín-Cordero, Dolores Muñoz-Mingarro, Antonio J. León-González, Richard B. Parsons, Nuria Acero

**Affiliations:** 1 Pharmaceutical and Health Science Department, Pharmacy Faculty, San Pablo-CEU University, CEU Universities, Urbanización Montepríncipe Boadilla del Monte, 28660 Madrid, Spain; a.canoyra@usp.ceu.es; 2 Department of Pharmacology, Faculty of Pharmacy, University of Seville, C/P. García González, 2, 41012 Seville, Spain; carmenmc@us.es (C.M.-C.); ajleon@us.es (A.J.L.-G.); 3 Chemistry and Biochemistry Department, Pharmacy Faculty, San Pablo-CEU University, CEU Universities, Urbanización Montepríncipe, Boadilla del Monte, 28668 Madrid, Spain; dmumin@ceu.es; 4 King’s College London, Institute of Pharmaceutical Sciences, 150 Stamford Street, London SE1 9NH, UK; richard.parsons@kcl.ac.uk

**Keywords:** *Corema album*, oxidative stress, neuroprotective, berries, phenols, Alzheimer’s disease, Parkinson’s disease

## Abstract

**Background/Objectives**: *Corema album* berries are edible fruits from the Iberian Atlantic coast, characterized by a rich polyphenolic composition, which endows their juice with potential protective effects against neurodegeneration. This study aimed to evaluate the potential of the relatively lesser-known *C. album* berries as a novel neuroprotective agent against neurodegenerative diseases. **Methods**: The phenolic compounds of the juice were characterized using UHPLC-HRMS (Orbitrap). The SH-SY5Y neuroblastoma line was used to determine the preventive effect of the juice against H_2_O_2_-induced oxidative stress. Furthermore, neuronal cells were differentiated into dopaminergic and cholinergic lines and exposed to 6-hydroxydopamine and okadaic acid, respectively, to simulate in vitro models of Parkinson’s disease and Alzheimer’s disease. The ability of the juice to enhance neuronal viability under toxic conditions was examined. Additionally, its inhibitory effects on neuroprotective-related enzymes, including MAO-A and MAO-B, were assessed in vitro. **Results:** Phytochemical characterization reveals that 5-*O*-caffeoylquinic acid constitutes 80% of the total phenolic compounds. Higher concentrations of the juice effectively protected both differentiated and undifferentiated SH-SY5Y cells from H_2_O_2_-induced oxidative damage, reducing oxidative stress by approximately 20% and suggesting a dose-dependent mechanism. Moreover, the presence of the juice significantly enhanced the viability of dopaminergic and cholinergic cells exposed to neurotoxic agents. In vitro, the juice inhibited the activity of MAO-A (IC_50_ = 87.21 µg/mL) and MAO-B (IC_50_ = 56.50 µg/mL). **Conclusions**: While these findings highlight *C. album* berries as a promising neuroprotective agent, further research is required to elucidate its neuroprotective mechanisms in cell and animal models and, ultimately, in human trials.

## 1. Introduction

Neurodegenerative diseases represent a significant global burden, and the incidence of these pathologies keeps growing. Parkinson’s disease and Alzheimer’s disease (PD and AD) are the most common age-related neurodegenerative disorders. Current treatments have not demonstrated a significant ability to prevent the progression of these diseases, highlighting the urgent need for the development of preventive and therapeutic agents. In this context, natural products and their active compounds are emerging as promising therapeutic candidates, which has resulted in a substantial increase in research interest in recent years [1].

Amongst other causes, neuronal degeneration is strongly correlated with oxidative stress, which is an early response in chronic neurodegenerative diseases. Consequently, up to 90% of the vulnerable neuronal population can be lost [2]. A wide variety of cellular macromolecules are affected by oxidative stress, suffering alterations that lead to neuronal dysfunction. Oxyradical-mediated modifications have been stated as one of the most common factors in the development of early-stage neurodegeneration. The brain is especially susceptible to oxidative stress because of its high oxygen consumption and relatively low levels of antioxidant compounds, and the large quantity of polyunsaturated fatty acids present in its tissues [3]. In addition, high levels of ROS increase the permeability of the blood–brain barrier, which leads to neurodegeneration and neuroinflammation [4]. For years, the consumption of antioxidants has been proposed as a potential preventive measure against oxidative damage. In this regard, polyphenol consumption is especially notable in the prevention of neurodegenerative disorders, as certain polyphenols have been shown to cross the blood–brain barrier, reaching neuronal cells [5]. Thus, the development of diseases such as PD, AD, and other dementias associated with age could be prevented. In fact, a diet enriched with polyphenols derived from berries, or even berry juice, has been shown to improve memory, particularly in elderly individuals [6,7].

Phytochemicals are well-known for their antioxidant properties, and although no current medications can halt the progression of neurodegenerative disorders, the potential neuroprotective effects of polyphenol antioxidants have been demonstrated. The neuroprotective capacity of polyphenols offers a new perspective for the treatment of neurodegenerative diseases, highlighting the role of oxidative stress in neurotoxicity and suggesting that phytochemicals may serve as potential protective agents against such conditions.

Clinical studies indicate that resveratrol is effective in lowering biomarkers linked to AD and brain ischemic stroke. Additionally, it has demonstrated adequate bioavailability at the administered dosages, with no significant adverse effects reported [8]. Although human clinical trials remain limited, preclinical research suggests that quercetin, hesperidin, genistein, and their flavonoids are promising and safe dietary compounds for the prevention of neurodegenerative diseases and the mitigation of their harmful effects. These molecules, therefore, warrant further investigation as potential complementary therapies for disorders such as PD and AD [9,10]. In this regard, Bellone et al. conducted a randomized trial that demonstrated pomegranate supplementation can enhance cognitive and functional recovery after ischemic stroke, a condition with an increased risk associated with PD [11].

Epidemiological studies on the relationship between polyphenol intake and PD are scarce, though some notable findings have emerged. A substantial prospective study conducted over two decades, involving nearly 130,000 participants, found that men with a high regular consumption of flavonoid-rich foods and beverages—such as tea—had a reduced risk of developing PD. This association was particularly evident for a higher intake of anthocyanins and anthocyanin-rich foods, such as berries and apples, which were linked to a lower risk of PD [12,13].

Monoamine oxidase (MAO) enzymes play a critical role in neurodegenerative diseases, including PD and AD. In the early stages of certain neurodegenerative processes, the activity and RNA expression of both MAO-A and MAO-B are significantly increased [14]. The link between MAO hyperactivity and amyloid-beta (Aβ) fibrillogenesis in AD has been demonstrated [15]. Furthermore, MAO-B inhibitors have the potential to prevent dopaminergic neuron degeneration and decrease symptoms of PD [16]. On the other hand, MAO-A inhibitors are considered a valuable treatment for anxiety and depression [17]. Given the high interest of natural plant-derived products in the pharmaceutical industry, as well as their therapeutic potential, research efforts are increasingly focused on identifying promising MAO inhibitors from natural sources due to their neuroprotective effects [18,19].

*Corema album* (L.) D.Don (Ericaceae) is an endemic shrub that grows on the coastal dunes of the Atlantic shoreline of the Iberian Peninsula. This dioecious evergreen understory shrub does not usually grow more than one meter in height [20]. Its fruits, measuring 5–10 mm in diameter, are white or pink-white berries with an acidic taste [21]. The berries of *C. album*, also known as white crowberries, pearlberries, beachberries or *camarinas*/*camariñas*/*camarinhas*, have been consumed for many centuries during the summer when they are fully ripe [22]. Historically, they have been consumed raw, as well as in liquors, lemonades, and jellies [23]. In traditional medicine, *C. album* has been used for its therapeutic effects against intestinal pinworm infection and fever [20,22,23,24].

Recent studies have highlighted the health benefits of *C. album* in relation to various diseases, including cancer, neurological, and cardiovascular diseases. The health-promoting properties of *C. album* are frequently attributed to its antioxidant capacity as a free radical scavenger, as well as its ability to enhance non-enzymatic cellular antioxidant defenses [25]. Phenolic compounds, mainly phenolic acids and flavonoids, are believed to be responsible for many of the health benefits associated with *C. album* [25,26]. Additionally, the anti-inflammatory potential of these compounds through the modulation of inflammatory mediators and enzymes has been demonstrated [26]. The presence of ursolic and oleanolic acids, which possess reflective optical properties, justify the interest in their use as natural sunscreens [27].

Despite its therapeutic potential, the consumption of this plant is relatively low, partly because it has never been commercially cultivated. Furthermore, the population of *C. album* has declined due to factors such as climate change and urban pressure [28]. For these reasons, it is important to increase the knowledge of this plant, which holds significant cultural and historical value in the Iberian Peninsula, not only to support scientific research that validates its therapeutic use but also to contribute to its conservation [29].

The purpose of this study was to investigate the potential neuroprotective properties of *C. album* berry juice. To this end, the chemical composition of the juice was analyzed using UHPLC-HRMS enabling the identification of its key components. The capacity of the juice to scavenge free radicals in vitro, as well as its ability to inhibit enzymes associated with neurodegenerative diseases, including MAO-A, MAO-B, and acetylcholinesterase (AChE), were measured. Furthermore, both the direct effects and the protective effects of the juice against oxidative stress were assessed in SH-SY5Y neuronal cell cultures, under normal culture conditions and following exposure to H_2_O_2_-induced oxidative stress. Finally, the ability of the juice to enhance neuronal viability in the presence of neurotoxic agents (6-hydroxydopamine (6-OHDA) and okadaic acid (OA)) was evaluated in cell culture models of PD and AD.

## 2. Results

### 2.1. Determination of Total Phenolic Compounds

The yield of the juice powder was 4.5% of the fresh berry weight. The total phenolic content was 63.45 ± 0.05 mg GAE/g (gallic acid equivalents/g) of juice powder, representing 6.3% of the total phenolic compounds. This value is significantly higher than the 1.3% of the total phenols we previously found by LC/MS in the aqueous extract or juice powder [30]. Therefore, further investigation into other water-soluble phenolic compounds, including proanthocyanidins and ellagic tannins, is necessary. Additionally, the juice is likely to contain other water-soluble compounds, such as vitamin C, sugars, and organic acids [25].

### 2.2. Determination of Flavonoids

Flavonoid quantification was performed using the AlCl_3_ reagent with (−)-epicatechin as the standard. The flavonoid content was 3.25 ± 0.01 mg (−)-epicatechin/g of juice powder, representing 5.1% of the total phenols. This result is comparable to the 6.7% obtained in our previous study on the aqueous extract using LC/MS [30].

### 2.3. Identification of Phenolic Compounds in Juice

UHPLC-HRMS (Orbitrap) was employed for the characterization of phenolic compounds in the juice. Several parameters, including chromatographic retention times, HRMS spectra, and MS/HRMS product ion scan spectra, were defined, with the data presented in Table 1. While some coelutions were observed among the phenolic compounds under analysis, they were effectively separated due to the high-resolution capability of the Q-Exactive Orbitrap HRMS instrument. The results are consistent with previously published data from our study on a water extract of *C. album* berries, analyzed by LC/MS [30].

Table 1 displays the identified compounds together with their retention times (RT), molecular formula, calculated [M − H]^−^ values, experimental [M − H]^−^ values, corresponding fragment ions, assignments, and accurate mass error (ppm) between the measured mass and the exact mass of each phenolic compound.

**Figure 1:**
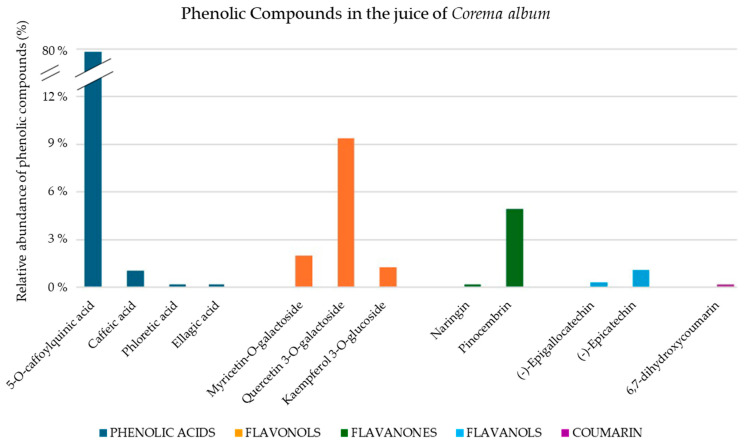
Relative abundance of phenolic compounds in powder juice of *Corema album* berries.

Twelve phenolic compounds were identified (Table 1), including four hydroxycinnamic acids (5-*O*-caffeoylquinic acid (5-CQA), caffeic acid, phloretic acid, and ellagic acid), three flavonols (myricetin-3-*O*-galactoside, quercetin-3-*O*-galactoside or hyperoside, and kaempferol-3-*O*-glucoside), two flavanones (naringin and pinocembrin), two flavanols ((−)-epigallocatechin and (−)-epicatechin), and one coumarin (6,7-dihydroxycoumarin or esculetin). Of these, four were identified for the first time in *C. album* berries: phloretic acid, ellagic acid, (−)-epigallocatechin, and 6,7-dihydroxycoumarin or esculetin. In this study, we confirmed the structures of quercetin-3-*O*-galactoside and myricetin-3-*O*-galactoside, which we were unable to confirm previously using HPLC, mass spectrometry, diode-array detection, and electrospray ionization–time-of-flight mass spectrometry [30]. Figure 1 illustrates the relative abundance (%) of the identified phenolic compounds.

The results show that hydroxycinnamic acids represent the predominant phenolic fraction in the juice powder, accounting for approximately 80% of the total phenolic compounds identified (Figure 1). Therefore, the biological effects of the extracts are most likely attributed to the group of hydroxycinnamic acids, particularly 5-CQA.

### 2.4. Radical Scavenging Capability In Vitro

#### 2.4.1. DPPH and ABTS Radical Scavenging Activities

The scavenging activity of white crowberries was determined by measuring 2,2-diphenyl-1-picrylhydrazyl (DPPH) free radicals (Table 2). The results suggest that the juice exhibited a slight antioxidant activity (IC_50_ = 412.7 ± 4.4 μg/mL) when compared to gallic acid (3.72 ± 0.02 µg/mL). Additionally, the scavenging capacity of the juice was assessed using 2,2′-azino-bis (3-ethylbenzothiazoline-6-sulfonic acid) diammonium salt (ABTS) radical cation. The ABTS scavenging activity of the juice was considerably higher compared to its DPPH scavenging capacity. However, the ABTS radical scavenging activity of the juice (IC_50_ = 112.4 ± 9.6 μg/mL) remained lower than that of the reference substance (IC_50_ = 28.6 ± 0.1 μg/mL).

#### 2.4.2. Hydroxyl Scavenging Activity

The hydroxyl radical scavenging activity of white crowberry juice was determined by calculating the percentage inhibition of hydroxyl radical formation in the Fenton reaction. The juice exhibited significantly higher hydroxyl radical scavenging activity (*p*-value = 0.039) than gallic acid (IC_50_ = 331 ± 2.1 μg/mL) (Table 2).

#### 2.4.3. Xanthine Oxidase Inhibition Assay

Xanthine oxidase is a flavoprotein that catalyzes the oxidation of hypoxanthine to xanthine and subsequently to uric acid while generating superoxide radicals in the process. White crowberry juice effectively scavenged superoxide radicals (IC_50_ = 426.9 ± 0.2 μg/mL) (Table 2). The decrease in superoxide radical levels observed in this assay may result either from radical scavenging or enzyme inhibition. To clarify this, the production of uric acid in the presence of juice or gallic acid was studied. The unaltered production of the final product of the reaction indicates that xanthine oxidase activity was unaffected by either the juice or gallic acid. Therefore, these results confirm the superoxide radical scavenging capacity of both the juice and the standard substance.

### 2.5. Neuroprotective Capacity: In Vitro Enzymatic Inhibition Capacity

#### 2.5.1. AChE Inhibition Assay

Galantamine is a well-known AChE inhibitor whose IC_50_ was determined to be 11.9 ± 1.8 μg/mL. In contrast, the juice of crowberries did not achieve AChE inhibition at the highest tested concentration (250 μg/mL). Therefore, these data do not support the classification of *C. album* juice as an effective inhibitor of this enzyme.

#### 2.5.2. MAO-A and MAO-B Inhibition Assay

Selegiline and clorgyline, which are recognized selective inhibitors of MAO-B and MAO-A, respectively, were used as positive controls in this experiment. The IC_50_ value for selegiline in inhibiting MAO-B was 49.55 ± 8.52 ng/mL, while the IC_50_ for clorgyline in inhibiting MAO-A was 5.04 ng/mL ± 0.33 ng/mL. The juice exhibited a significant, dose-dependent inhibitory activity against both enzymes, with IC_50_ values of 87.21 ± 0.01 µg/mL for MAO-A and 56.50 ± 0.006 µg/mL for MAO-B (Table 3).

### 2.6. Intracellular ROS Production

**Figure 2:**
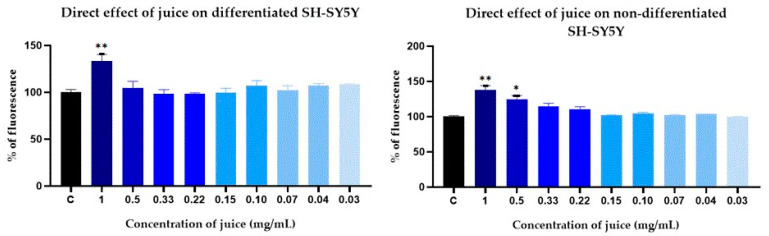
Intracellular ROS production in differentiated and non-differentiated SH-SY5Y cells after 90 min of exposure to *Corema album* juice. Results are expressed as percentage of fluorescence relative to untreated cells (control, in black) and are presented as the mean ± S.E. of *n* = 3. Significant differences, determined by one-way ANOVA–Bonferroni, are indicated with * *p* < 0.05 and ** *p* < 0.01.

The effect of the juice at various concentrations on the intracellular ROS levels was evaluated in cultured SH-SY5Y human neuroblastoma cells, both before and after differentiation with retinoic acid (RA) (Figure 2). The SH-SY5Y cell line is widely recognized as a useful neuronal model [31]. To assess the direct effect of *C. album* fruit, differentiated and non-differentiated SH-SY5Y cells were exposed to different concentrations of the juice. Following treatment, fluorescence was quantified. An increase in fluorescence corresponded to a rise in intracellular ROS levels. After 90 min of treatment, no significant differences were observed between the control and the tested concentrations, except at 1 mg/mL, the highest concentration studied. This concentration resulted in a significant increase in ROS levels compared to the control, regardless of the cells’ differentiation status. Additionally, non-differentiated cells exposed to 0.5 mg/mL of the juice resulted in a significant increase in ROS levels (Figure 2).

**Figure 3:**
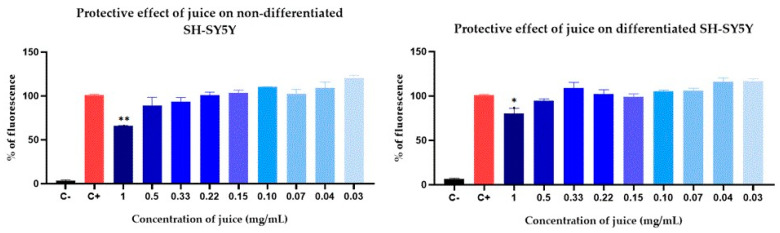
ROS production in differentiated and non-differentiated SH-SY5Y cells after 24 h incubation with *Corema album* berry juice followed by 90 min of exposure to H_2_O_2_. Results are expressed as percentage of fluorescence relative to untreated stressed cells (positive control, in red) and are presented as the mean ± SE of *n* = 3. Unstressed cells were used as negative control (in black). Significant differences, determined by one-way ANOVA–Bonferroni, are indicated with * *p* < 0.05 and ** *p* < 0.01.

The protective effect of *C. album* fruit juice against oxidative stress was also studied. Differentiated and non-differentiated SH-SY5Y cells were incubated with different concentrations of the juice for 24 h. Following incubation, 0.5 mM H_2_O_2_ was added to the medium, and the fluorescence was measured over 90 min to assess ROS levels. Two controls were included, neither of which were treated with the juice: a negative control (in black) consisting of unstressed cells and a positive control (in red) consisting of cells subjected to oxidative stress with hydrogen peroxide. The results presented in Figure 3 show a substantial increase in intracellular ROS levels after 90 min of oxidative stress induction compared to non-stressed cells. Cells pretreated with 1 mg/mL of the juice demonstrated significantly reduced ROS levels compared to the stressed control. No significant differences were observed between the other treatments and the positive control.

These results suggest that *C. album* berries may have protective effects on neuronal cell lines, being more prominent in the ones that are not differentiated.

### 2.7. Cell Viability: Parkinson’s Disease and Alzheimer’s Disease Models

**Figure 4:**
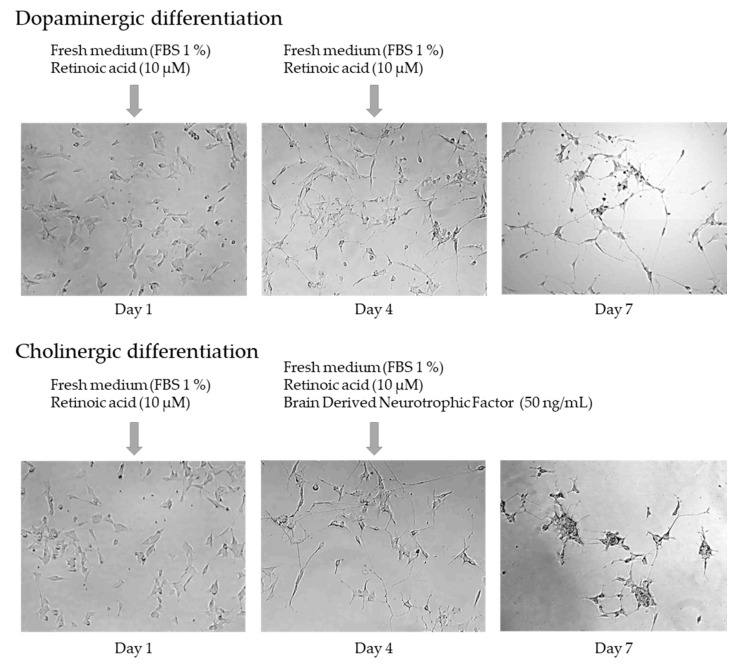
Differentiation protocols. Proliferative SH-SY5Y cells were seeded and cultured in medium supplemented with 1% FBS (fetal bovine serum) and RA (retinoic acid) (10 µM) for 3 days to induce differentiation. For dopaminergic differentiation, cells were supplemented with 1% FBS and RA (10 µM) on day 4. BDNF (brain-derived neurotrophic factor) (50 ng/mL) was added to cells to redirect differentiation toward cholinergic cells.

To evaluate the effects of the juice on different types of neuronal cells, the SH-SY5Y neuroblastoma line was differentiated into cholinergic and dopaminergic cell lines (Figure 4) and subjected to different concentrations of juice for 24 h.

**Figure 5:**
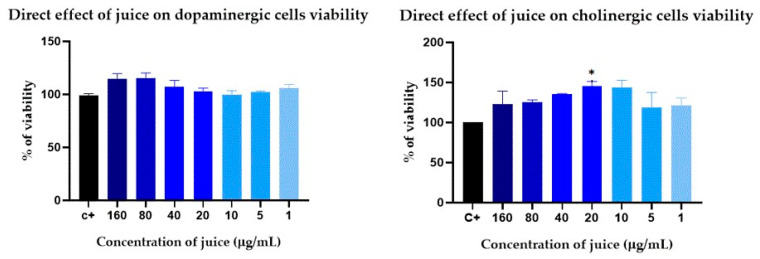
Direct effect of *Corema album* fruit juice in the viability of dopaminergic and cholinergic cells. Dopaminergic and cholinergic lines were exposed to different concentrations of *C. album* berry juice for 24 h. Results are expressed as percentage of viability relative to untreated cells (control, in black) and are presented as the mean ± SE of *n* = 4. Significant differences, determined by one-way ANOVA–Bonferroni, are indicated with * *p* < 0.05.

As shown in Figure 5, the juice did not affect dopaminergic cell viability at concentrations up to 160 μg/mL. However, cholinergic cells viability had a significant increase of 45.3 ± 3.4% after treatment with 20 μg/mL, with a slight, though not significant, increase in viability at other concentrations.

The results obtained highlight the beneficial effects of the juice on neuronal cell lines, promoting the viability and health of neuronal cells while potentially preventing the early onset of AD.

Based on these results, the protective effect of the juice was evaluated in models of PD and AD.

Dopaminergic cells were incubated for 24 h with juice (80 μg/mL) and different concentrations of 6-OHDA. Likewise, cholinergic cells were incubated for 24 h with juice (20 μg/mL) and various concentrations of OA. After incubation, MTT assays were performed to assess cell viability, which was compared with the control group that had not been exposed to the juice.

**Figure 6:**
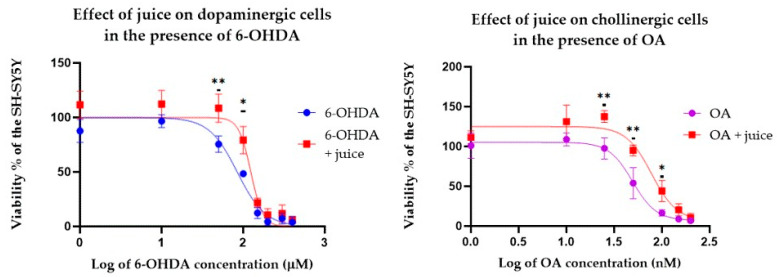
Protective effect of *Corema album* fruit juice on the viability of dopaminergic and cholinergic cells in the presence of 6-hydroxydopamine (6-OHDA) or okadaic acid (OA), respectively. Cells were treated with *C. album* juice for 24 h, followed by the addition of different concentrations of 6-OHDA or OA. The results are expressed as the percentage of viability relative to control cells treated with the juice but not with the toxic substance and are presented as the mean ± SE (*n* = 4). Significant differences, determined by two-way ANOVA–Bonferroni, are indicated with * *p* < 0.05) or ** *p* < 0.01.

Figure 6 illustrates the dose–response curves for dopaminergic and cholinergic cells exposed to 6-OHDA and OA, as appropriate, in the presence and absence of juice. The presence of juice significantly enhanced neuronal survival in both models. 6-OHDA exhibited an LC_50_ of 124.10 ± 6.68 μM in dopaminergic cells, which decreased to 84.51 ± 4.39 μM with the addition of the juice. On the other hand, OA had an LC_50_ of 98.71 ± 11.38 μM in cholinergic cells, which decreased to 56.29 ± 4.58 μM in the presence of juice. Furthermore, incubation with juice increased the viability of cells at individual concentrations of the toxin in each disease model. Thus, the results suggest that juice may be protective against the onset of PD and AD.

## 3. Discussion

Research into novel natural products with the potential to prevent or delay neurodegeneration is a major challenge. *C. album* berries have demonstrated beneficial health effects, primarily attributed to their antioxidant properties. The high antioxidant capacity has been related to juice’s rich content of phenolic compounds [32,33].

Notably, it contains hydroxycinnamic acids, such as 5-CQA; flavonols, such as myricetin 3-*O*-galactoside, quercetin 3-*O*-galactoside, and myricetin 3-*O*-glucoside; flavanones, such as naringin and pinocembrin; flavanols, such as (−)-epicatechin and (−)-epigallocatechin; and a coumarin, 6,7-dihydroxycoumarin (esculetin). Our findings suggest that the juice from *C. album* fruits exhibits antioxidant properties. Since oxidative stress is a key factor in the pathogenesis of neurodegenerative diseases [34], it is hypothesized that increasing antioxidant intake may help prevent or mitigate these conditions. In this context, the ability of various polyphenols to cross the blood–brain barrier is crucial [5]. Preclinical studies have demonstrated that flavonoids can have significant effects on mammalian cognitive function, potentially reversing age-related declines in memory and learning. Clinical trials on this topic remain limited; however, several epidemiological studies have investigated the impact of certain fruits, including strawberry, bilberry, blackcurrant, blueberry, and blackberry, on brain health [35]. However, as other authors have recently noted, despite the wide variety of phenolic compounds in *C. album* juice, its therapeutic potential and health benefits have been scarcely investigated [25]. In light of our results and considering that the neuroprotective properties of these fruits are attributed to phytochemicals, like caffeic acid, catechin, quercetin, and kaempferol—all of which are present in the juice examined in this study—our findings are particularly promising. Nutritional interventions rich in phytochemicals, such as the consumption of berries or the moderate intake of beverages, like tea, coffee, and red wine, have been linked to improved mental health outcomes and could serve as valuable tools in preventing aging-related cognitive decline or delaying the onset of neurodegenerative diseases [35,36].

Previous studies have analyzed the composition of fruit and leaf extracts of *C. album* and their ability to prevent inflammation, oxidative stress, and carcinogenesis [21,25]. For example, León et al. demonstrated the capacity of white crowberry juice extracts in human hepatocellular carcinoma (HepG2) cell cultures, not only as ROS scavengers but also in enhancing cellular antioxidant defenses in this cell line [30].

In this study, we initially assessed the free radical scavenging activities of *C. album* juice in vitro. The juice exhibits considerable phytochemical complexity, making it necessary to employ more than a single type of in vitro assay to accurately evaluate its antioxidant activity. Each method varies in terms of radical generation, sensitivity, the specific antioxidant properties measured, and the approach used to assess the inhibition reaction’s endpoint. Additionally, the inclusion of reference compounds, such as gallic acid, is essential for comparative purposes [37]. The juice showed a moderate free radical scavenging capacity compared to the reference substance in most of the radical assays studied (Table 2). However, in relation to its ability to capture hydroxyl radicals, it showed even greater effectiveness than gallic acid, with IC_50_ of 221.2 µg/mL and 331.4 µg/mL, respectively.

Oxygen-derived free radicals may play a critical role in the initiation and/or progression of PD and AD, ultimately contributing to neuronal cell death [38]. Moreover, the release of ROS during dopamine oxidation by MAO-B in dopaminergic neurons has been associated with increased oxidative stress in specific brain regions, which is proposed to contribute to the pathogenesis of PD [39]. Several factors, such as brain trauma and aging, are known to enhance free radical formation, and these factors are recognized as risk factors for Alzheimer’s-type dementias. Free radicals, especially hydroxyl radicals, can induce cell death through lipid and protein damage, while their interactions with neurotransmitters may lead to the production of endogenous neurotoxins [40]. Extensive research has investigated the oxidative effects of hydroxyl radicals on membrane lipids, with polyunsaturated fatty acids (PUFAs) being particularly prone to oxidation. The peroxidation of PUFAs by hydroxyl radicals is one of the most detrimental attacks on cellular integrity. The brain is especially rich in PUFAs, making it highly susceptible to ROS-induced damage [41]. Thus, our results suggest that crowberry juice may be a useful antioxidant source to prevent oxidative-induced neurodegeneration.

Phenolic compounds, among secondary plant metabolites, represent some of the most significant naturally occurring antioxidants. The number of hydroxyl groups in their chemical structure plays a crucial role in determining their antioxidant capacity [42]. UHPLC-HRMS analysis reveals that the major components of the juice are phenolic acids and flavonols. Previous studies have reported the antioxidant activities of phenolic compounds, such as caffeic acid and quercetin-3-*O*-galactoside [43]. The radical scavenging activity of the juice may be attributed to these components [44,45].

The capacity of the juice to inhibit MAO-A and MAO-B enzymes was analyzed, and the results were compared to those of clorgyline and selegiline, which are selective inhibitors of these enzymes [46]. MAO-A preferentially oxidizes serotonin (5-hydroxytryptamine) and norepinephrine, whereas MAO-B primarily oxidizes phenylethylamine. Dopamine can be oxidized by both isoforms [47,48]. MAO-B is implicated in the neurodegenerative processes associated with aging and is involved in diseases such as PD and AD. MAO-B inhibitors, which have been shown to possess neuroprotective effects, can prevent the degeneration of dopaminergic neurons and reduce the production of neurotoxins [49]. In contrast, while MAO-A is primarily associated with depression, recent evidence suggests that this enzyme is directly involved in neuronal loss in PD and other neurodegenerative disorders through the activation of mitochondrial death signaling pathways [50].

A wide range of plant extracts, including foods and herbal remedies, has been investigated for their potential to inhibit MAO [25]. Research has revealed many plant-derived compounds, such as quercetin, capable of inhibiting one or both isoforms of MAO [51]. Other flavonoids present in the juice show selective inhibition of MAO-B, such as (−)-epicatechin and naringenin, while kaempferol has been identified as a more potent inhibitor of MAO-A [52].

Among hydroxycinnamic acids, 5-CQA, one of the most abundant phenolic acids in *C. album* juice, has also been reported to inhibit both MAO-B [53] and MAO-A isoforms [54]. This compound has also been shown to possess notable neuroprotective properties via several mechanisms, particularly through its ability to mitigate oxidative stress. For example, it has been found to decrease apoptosis in primary cortical neurons by enhancing the expression of antioxidant enzymes, such as NAD(P)H quinone oxidoreductase 1 [55]. Additionally, 5-CQA has been reported to protect murine adrenal cells from apoptosis triggered by hydrogen peroxide by preventing mitochondrial membrane depolarization induced by oxidative stress [56]. Furthermore, 5-CQA has been observed to defend HepG2 liver cancer cells against reactive oxygen species generated by t-butyl hydroperoxide [30]. On the other hand, berry anthocyanidins, such as cyanidin-3-glucoside, have also demonstrated MAO inhibitory activity, with IC_50_ values in the low micromolar range [57]. Although not identified in this study, these types of compounds have been previously described in *C. album* juice by León et al. [30]. It is plausible that the neuroprotective activity of the whole juice results from the combined, additive, or synergistic effects of multiple metabolites, rather than the action of a single dominant compound. This suggests that the complex phytochemical profile of the juice can provide health benefits that cannot be replicated by isolated molecules [52].

Current research underscores the potential of natural compounds in promoting human health, with the wide diversity of secondary metabolites positioning them as a valuable source of novel therapeutic agents [58]. Increasing interest is focused on the use of secondary metabolites, such as polyphenolic compounds, in the prevention and management of neurodegenerative diseases. These secondary metabolites, with their broad range of beneficial effects on neurological health, merit particular attention due to their capacity to target multiple pathways simultaneously, offering potential therapeutic benefits for disorders with complex pathophysiologies [59]. Moreover, additional characteristics of structurally and functionally diverse secondary metabolites, such as their ability to cross the blood–brain barrier, metabolism, required dosage for beneficial effects in humans, and their safety profile in terms of toxicity and interactions with current medications, are also crucial factors to consider.

Our results indicate that juice is a strong MAO inhibitor in vitro, capable of affecting both isoforms. To our knowledge, the inhibitory effect of *C. album* fruit juice on dopamine metabolism is reported here for the first time. If these results are confirmed in vivo studies, the juice may prove to be a promising dietary supplement for the management of neurodegenerative diseases.

To better understand the potential of the juice in reducing neurodegeneration, we also evaluated its effect on intracellular ROS production. The results indicate that direct exposure to the juice, particularly at the highest concentrations tested, led to an increase in ROS levels in neuronal cells (Figure 4). ROS plays an important role in physiological processes, including the regulation of cell signaling, cell growth, apoptosis, differentiation, and activity of several enzymes. It is known that a slight increase in ROS production can trigger the activation of antioxidant defense mechanisms within the cell, thereby offering protection against oxidative damage [55,56,60]. As shown in Figure 5, the juice at higher concentrations significantly reduces oxidative stress induced by H_2_O_2_. Based on our findings, the juice-induced ROS production may activate antioxidant mechanisms, which become evident when cells are exposed to oxidative stress.

Additionally, in human neuronal cells, neurotoxins, such as 6-OHDA and OA, produce neuronal loss, leading to the appearance and development of neurodegenerative diseases [61,62]. The high concentrations of 5-CQA in *C. album* fruit juice, whose neuroprotective activity has been discussed above, may explain its preventive effects against toxin-induced damage in dopaminergic and cholinergic cells, underscoring the need for further research in this area. We found that white crowberry juice increased neuronal viability in the presence of neurotoxic agents, which, if replicated in vivo, suggests that it may delay the emergence and progression of neurodegeneration. Although further research is needed, the protective effect of the juice on neuronal viability in PD and AD may be given by its antioxidant effect and/or ability to effectively inhibit MAO-A and MAO-B.

Finally, the protective effect displayed by the juice against oxidative damage by inhibiting the production of intracellular ROS was more pronounced in non-differentiated cells. These ‘immature’ SH-SY5Y neurons are frequently used as a neuronal model to mimic the neurons present in infants [63]. As previously mentioned, neurodegenerative diseases such as AD and PD develop over the course of a lifetime, although symptoms typically appear in later stages of life. Therefore, proper neuroprotection is of vital importance from childhood onward. Our results indicate the potential benefits of administering this juice from early childhood to enhance neuronal function and viability, supporting neuronal development during this critical period. It also may explain the long-term health benefits of consuming berries throughout life. This effect could promote the proper functioning and complete maturation of neurons, potentially reducing the onset and progression of neurodegenerative diseases. Natural products or plant-derived antioxidant extracts could be a promising option for pre-symptom treatment, as they are generally well-tolerated, infrequently associated with side effects, and widely accepted by the population [64].

To investigate the role of free radical-induced damage in PD and AD, it is crucial to develop appropriate animal models that can facilitate the screening and evaluation of novel therapeutic approaches targeting the underlying pathogenic mechanisms. In vitro studies employing various toxin-induced cell lines, such as SH-SY5Y (used in this study), as well as others, like PC12 and MN9D, replicate the key aspects of dopaminergic neuron degeneration seen in PD [65]. Our results should be cautiously interpreted, as they were derived from in vitro cell-based assays; however, we believe that the promising data obtained deserves to be highlighted. Consequently, additional studies incorporating these PD and AD animal and cellular models are essential to further elucidate the mechanisms of action and assess the neuroprotective potential of *C. album* fruits, thereby supporting the transition from in vitro to therapeutic use.

## 4. Materials and Methods

### 4.1. Plant Material and Extraction of Juice from Berries of C. album

Wild *C. album* berries were collected in Huelva (Spain) (37°04′10.15″ N–6°41′15.45″ W) in September 2022. The identification was carried out by Dr. Maria Cruz Diaz Barradas from the Department of Plant Biology and Ecology, University of Seville. The ripe fruits were frozen and stored at −20 °C until further analysis. To obtain the juice, 300 g of frozen berries were homogenized with 300 mL of water using ultrasonic equipment for 45 min at room temperature. The resulting water extract was filtered and lyophilized and yielded 4.5% of juice powered with respect to fresh fruit weight. The dried extract was then prepared for phenolic composition analysis via UHPLC/HRMS and various assays.

### 4.2. Reagents

Formic acid (98%) and HPLC grade methanol were obtained from Panreac (Barcelona, Spain). 5-*O*-caffeoylquinic acid (≥98%), caffeic acid (≥98%), phloretic acid (≥98%), ellagic acid (≥95%), gallic acid (≥98.5%), 6,7-dihydroxycoumarin (≥98%), and Folin–Ciocalteau reagent were purchased from Sigma-Aldrich (Madrid, Spain). Myricetin-3-*O*-galactoside (≥99%), quercetin 3-*O*-galactoside (≥98%), kaempferol-3-*O*-glucoside (≥99%), naringin (≥99%), pinocembrin (≥98%), (−)-epigallocatechin (≥98%), and (−)-epicatechin (≥98%) were acquired from Extrasynthese (Lyon, France).

### 4.3. Determination of Total Phenolic Content

The total phenolic content of the juice powder was measured using the Folin–Ciocalteu method [66] and expressed as milligrams of gallic acid equivalents (GAE) per gram of dry weight or powder juice (mg GAE/g DW).

### 4.4. Determination of Total Flavonoids

Flavonoid determination was carried out using the colorimetric assay developed by Lamaison and Carnat, adapted to a microplate format [67]. The results are expressed as mg of (−)-epicatechin per gram of dry weight.

### 4.5. Analytical Instrumentation

Chromatographic separation was performed on a UHPLC Dionex Ultimate 3000 RS system (Thermo Fisher Scientific, San Jose, CA, USA), which included a binary pump, autosampler, and column oven. An Acquity BEH C18 reversed-phase column (particle size 1.7 µm, 2.1 × 100 mm) from Waters (Milford, MA, USA) was used for the analysis. The separation was carried out using gradient elution with 0.1% formic acid in water (solvent A) and methanol containing 0.1% formic acid (solvent B). The gradient program was as follows: 0–1 min, isocratic conditions at 5% B; 1–10 min, linear gradient from 5% to 10% B; 10–12 min, isocratic at 100% B; and 12–15 min return to initial conditions at 5% B to re-equilibrate the column. The mobile phase flow rate was 0.5 mL/min, and the injection volume was 5 μL. The UHPLC system was coupled to a Q-Exactive Orbitrap HRMS (Thermo Fisher Scientific, San Jose, CA, USA) with a heated electrospray ionization (HESI-II) source operating in negative ionization mode. Nitrogen was used as sheath gas, sweep gas, and auxiliary gas at flow rates of 60, 0, and 25 arbitrary units (au), respectively. The HESI-II heater temperature was set to 400 °C, with a capillary voltage of −3.0 kV. The instrument’s capillary temperature was 320 °C, and an SLens RF level of 50 V was applied. The Q-Exactive Orbitrap HRMS was calibrated and tuned every 7 days using Thermo Fisher’s commercial calibration solution. The HRMS was operated in full MS scan mode with a mass range from 50 to 750 *m*/*z* and a resolution of 70,000 full width at half-maximum (FWHM) at *m*/*z* 200 with an automatic gain control (AGC) target set to 3.0 × 10^6^ ions, with a maximum injection time (IT) of 200 ms. Following the full MS scan, data-dependent scans were performed in product ion scan mode (Top5), with fragmentation applied at stepped normalized collision energies (NCE) of 30, 60, and 90 eV. Product ion spectra were recorded with an isolation window of 0.7 *m*/*z*. For this stage, the mass resolution was 17,500 FWHM at *m*/*z* 200, with an AGC target of 2.0 × 10^5^ and a maximum IT of 50 ms. Data-dependent scans were triggered with an intensity threshold of 1.6 × 10^5^.

Instrument control and data acquisition were managed using Xcalibur v 4.3 software, while data analysis was performed with Trace Finder v 5.1 software (Thermo Fisher Scientific, San Jose, CA, USA). Identification was achieved by comparing retention times, the exact masses of the pseudomolecular ions, and their fragment ions (with a maximum deviation of 5 ppm) against a database of phenolic and phytohormone compounds. Additionally, isotopic pattern match scores greater than 80% were required for confirmation.

### 4.6. In Vitro Radical Scavenging Assays

#### 4.6.1. DPPH Scavenging Assay

A 2,2-diphenyl-β-picrylhydrazyl (DPPH) assay was used to evaluate the white crowberry’s potential to scavenge free radicals in vitro [68]. In this assay, the juice capability to reduce DPPH radical was measured. The DPPH changes color because of this reduction, changing from purple (the oxidized form) to yellow (the reduced form) with an absorption maximum of 540 nm. A total of 200 µL of MeOH, 50 µL of DPPH 0.022% in MetOH, and 30 µL of juice or gallic acid at different concentrations were mixed in a 96-well plate. For control samples, 250 µL of MeOH and 50 µL of DPPH 0.022% were added. The absorbance of the plate was measured in a SpectroStar Nano (BMG Labtech, Ortenberg, Germany) plate reader at 540 nm, after being incubated for 20 min at room temperature in the dark. The reduction percentage of DPPH was computed using the following formula:

Reduction % = [(Abs_0_ − Abs_1_)/Abs_0_] × 100 (1)

where Abs_0_ represents the control’s absorbance, and Abs_1_ indicates the ascorbic acid or juice absorbance at each concentration. Using this information, the concentration at which the DPPH radical could be reduced by 50% was determined to be the IC_50_ for each drug. The mean IC_50_ of the three replicates was used to express the results.

#### 4.6.2. ABTS Assay

The 2,2′-azinobis-(3-ethylbenzthiazoline)-6-sulfonic acid ABTS radical scavenging assay measures the extract’s ability to scavenge this radical [69].

The experiment was carried out in a 48-well plate, and the reagents were prepared in phosphate-buffered saline (PBS). The following reagents were added to each well: 228 μL of water, 400 μL of ABTS (500 μM), 12 μL of myoglobin III (myoglobin 400 μM: K_4_Fe(CN)_6_ 740 μM, 1:1), 20 μL of juice/standard/PBS at various concentrations, and 340 μL of H_2_O_2_ (450 μM) right before measuring. The extract and standard concentrations, dissolved in PBS, were found to range from 1 to 0.04 mg/mL and 50 to 2.9 μg/mL, respectively. After 15 min of incubation, absorbances were measured at 734 nm using a Spectrostar Nano plate reader (BMG Labtech, Ortenberg, Germany). Every assay was run in triplicate. Following the acquisition of the data, the radical reduction percentage and the IC_50_ were computed using the following equation:

Reduction % = [(Abs_0_ − Abs_1_)/Abs_0_] × 100 (2)

The absorbance from the control, Abs_0_, was compared to the absorbance from the juice or the standard (gallic acid) at each concentration (Abs_1_). The concentration of juice or standard that can lower the ABTS radical by 50% is known as the IC_50_.

#### 4.6.3. Xanthine/Xanthine Oxidase Assay

Superoxide radicals are produced by the xanthine/xanthine oxidase system and have the capacity to reduce NBT (nitroblue tetrazolium). Antioxidants can capture these free radicals and stop NBT from reducing. The absorbance changes at 560 nm over time were used to quantify the white crowberry´s juice capture capacity at different doses [70].

Gallic acid was used as the reference substance. Every reagent was prepared in phosphate buffer (pH 7.4, 50 mM KH_2_PO_4_/KOH). In each well, 10 µL of EDTA (ethylenediaminetetraacetic acid) (15 mM), 15 µL of hypoxanthine (3 mM), and 25 µL of NBT 0.6 mM were added. For the negative control, xanthine oxidase was not added. For the positive control and samples, 25 µL of xanthine oxidase (0.1 U/mL) and 12.5 µL of juice/gallic acid at various concentrations were added. Finally, phosphate buffer was added to every well, reaching a final volume of 150 µL. Using a Spectrostar Nano plate reader (BMG Labtech, Ortenberg, Germany), absorbance measurements were performed for 40 min at intervals of 5 min at a wavelength of 560 nm. The results are presented as a percentage of NTB reduction inhibition relative to the control.

The amount of uric acid produced (295 nm) was also measured to assess the capacity to inhibit the enzyme xanthine oxidase. The findings are presented as a percentage of the enzyme xanthine oxidase’s inhibition. Every test was run in triplicate.

#### 4.6.4. Hydroxyl Radical Scavenging Capacity

The reaction mixture for the assessment of the hydroxyl scavenging capacity of the juice included 1 mL of FeSO_4_ 1.5 mM, 0.7 mL of H_2_O_2_ 6 mM, 0.3 mL of sodium salicylate 20 mM, and 1 mL of various concentrations of the juice or gallic acid to determine the hydroxyl radical scavenging capacity of white crowberry juice and a reference substance [71,72]. Using the SpectroStar Nano (BMG Labtech, Ortenberg, Germany) plate reader, the hydroxyl complex absorbance was measured at 562 nm following a 1 h incubation at 37 °C. The following equation was used to obtain the scavenging % capacity:

% scavenging capacity = [1 − (Abs_1_ − Abs_2_)/Abs_0_] × 100 (3)

where Abs_0_ represents the control’s absorbance, Abs_1_ represents the juice’s or the reference compound’s absorbance, and Abs_2_ represents the juice’s or the reference compound’s absorbance without sodium salicylate. All tests were conducted in triplicate.

### 4.7. Neuroprotective Effect on Enzymes In Vitro

#### 4.7.1. MAO-A and MAO-B Inhibition Assay

A 96-well plate was used for the assay, following the procedure described by Burgos et al. [73]. In each well, 50 µL of juice at various concentrations dissolved in dimethyl sulfoxide (DMSO), 50 µL of clorgyline or selegiline as reference compounds, or 50 µL of DMSO as a control, were mixed with 50 µL of chromogenic solution (0.8 mM vanillic acid, 0.417 mM 4-aminoantipyrine, and 4 U/mL peroxidase in 0.2 M potassium phosphate buffer, pH 7.6). Additionally, 100 µL of tyramine (3 mM in 0.2 M potassium phosphate buffer, pH 7.6) and 50 µL of MAO-A/MAO-B (8 U/mL in 0.2 M potassium phosphate buffer, pH 7.6) were added. Each test was performed in triplicate.

Absorbance measurements were taken every 5 min for 40 min at 37 °C using a SpectroStar Nano plate reader (BMG Labtech, Ortenberg, Germany). The IC_50_ values (the concentration required to inhibit 50% of enzyme activity) were determined for each substance.

#### 4.7.2. Acetylcholinesterase (AChE) Inhibition Assay

Acetylthiocholine was used in a colorimetric assay to assess the effect of crowberries on AChE activity. The enzymatic hydrolysis of acetylthiocholine produces thiocholine, which then reacts with DTNB (5,5′-dithio-bis-2-nitrobenzoate) to form yellow anion 5-thio-2-nitrobenzoic acid, measurable at 405 nm [74].

The reaction was carried out on a 96-well plate. Each well contained 25 µL of juice dissolved in 50 mM buffer at pH 8, galantamine (used as a positive control), or 50 mM pH 8 Tris-HCl buffer as a negative control; 25 µL of acetylthiocholine (15 mM) in Milli-Q water; 125 µL of 3 mM DTNB (5,5′-dithiobis-(2-nitrobenzoic acid)) in buffer C (Tris-HCl 50 mM pH 8, NaCl 0.1 M, and MgCl_2_ 0.02 M); and 50 µL of buffer B (50 mM pH 8) with 0.1% BSA (bovine serum albumin). After 30 s, initial absorbance was measured at 405 nm (T0), and then 25 µL of acetylcholinesterase (0.22 U/mL in A buffer) was added. Absorbance was measured every 90 s for a total of 15 min at 37 °C using the SpectroStar Nano plate reader (BMG Labtech, Ortenberg, Germany). All assays were performed in triplicate. The results are expressed as a percentage of enzyme inhibition for each juice or galantamine concentration.

### 4.8. Cellular Assays

#### 4.8.1. Cell Culture and Differentiation

The cell culture of the SH-SY5Y cell line was performed using the method described by de Medeiros et al. [75]. SH-SY5Y cells were seeded in 96-well plates at a density of 10^4^ cells/cm^2^ in a medium containing 1% heat-inactivated FBS (fetal bovine serum) and incubated for 24 h before differentiation. Dopaminergic neuronal differentiation was induced by adding 10µM retinoic acid (RA) (Enza Life Sciences, Lörach, Germany) to the medium for 7 days. The medium was refreshed every 3 days to maintain the appropriate concentration of RA. For cholinergic neuronal differentiation, the method of de Medeiros et al. [75] was followed On the fourth day of differentiation, the cells were further supplemented with 50 ng/mL of brain-derived neurotrophic factor (BDNF, Human Recombinant, Prospec^®^, East Brunswick, NJ, USA). RA was dissolved in absolute ethanol to prepare the stock solutions, while BDNF solution was prepared in 0.1% bovine serum albumin (BSA).

#### 4.8.2. Intracellular ROS Production Analysis

A 2,7-dichlorodihydrofluorescein diacetate (DCFH-DA) assay was employed to assess the intracellular ROS formation and evaluate the antioxidant capacity of white crowberry juice in cell cultures [76]. DCFH-DA enters cells, where it is deacetylated to form 2,7-dichlorodihydrofluorescein (DCFH). Upon oxidation by ROS, DCFH is converted into the fluorescent compound 2,7-dichlorofluorescein (DCF); thus, the fluorescence intensity is proportional to the amount of ROS present.

For the assay, 10^4^ SH-SY5Y cells were seeded in 96-well plates and incubated at 37 °C with 5% CO_2_ for 24 h. Following incubation, 100 µL of DCFH-DA 0.02 mM in PBS was added to each well to evaluate its direct effect. After a 30 min incubation, the cells were washed with PBS, and various concentrations of crowberry juice dissolved in the culture medium were added. Fluorescence measurements were taken immediately after the juice was added.

For a protective effect, cells were pre-treated with different concentrations of juice in a culture medium supplemented with 1% FBS for 24 h. Following pre-treatment, 0.02 mM DCFH-DA was added, and the plates were incubated for another 30 min. Afterward, the cells were washed with PBS, and 500 µM H_2_O_2_ was added to induce oxidative stress. Fluorescence measurements were started immediately after H_2_O_2_ addition. A Varioskan Lux (ThermoScientific, San Jose, CA, USA) plate reader was used to measure fluorescence every 15 min over 90 min, with an excitation wavelength of 485 nm and emission wavelength of 520 nm. The results are expressed as the percentage of fluorescence relative to the untreated stressed cells (positive control) after 90 min of H_2_O_2_ exposition. All experiments were conducted in triplicate.

#### 4.8.3. Cytotoxic and Neuroprotective Effect Assay

The direct and protective effects of the juice against dopaminergic and cholinergic cell lines were studied. To evaluate the direct effect, both cell lines were exposed to different concentrations of juice (160–0 µg/mL) for 24 h, after which the viability of the cells was measured.

To assess the protective effect of the juice in the PD and AD models, the dopaminergic and cholinergic cell lines were separated into two groups. The first group was exposed to the juice for 24 h except for the control samples (80 µg/mL for the dopaminergic cells and 20 µg/mL for the cholinergic cells). The second group was treated with a regular medium. Following incubation, different concentrations of 6-OHDA were added to the dopaminergic cells (300–1 µM) in the PD model. Likewise, OA was added to the cholinergic cells (200–1 nM) to simulate AD. After 24 h of incubation, the viability of both cell lines was measured [63].

An MTT assay was performed to assess the cell viability of SHSY-5Y cells after being treated [77]. An MTT stock solution was prepared in PBS at a concentration of 6 mg/mL. Cells were washed with 100 µL PBS. Following washing, 10 µL of the 6 mg/mL MTT solution was added to each well except for the control wells. The plate was then briefly spun and incubated for 4 h at 37 °C and protected from the light. Following incubation, the media was carefully aspirated, and 100 µL of DMSO was added. The plate was gently spun before the absorbance of the wells was measured at 570 nm.

The results are expressed as the percentage of viability relative to the control cells treated with the juice but not with the toxic substance.

### 4.9. Statistical Analysis

Statistical analysis was conducted using GraphPad Prism 8.0.1. In vitro experiments were performed in triplicate, while cellular assays were conducted with *n* = 4. All data are presented as the mean ± standard error (SE). Statistical comparisons were made using one-way (in vitro tests and ROS assays) and two-way analysis of variance (ANOVA) (viability under toxic conditions), followed by the Bonferroni post hoc test. Differences were considered statistically significant (*) when the *p*-value was less than 0.05 and highly significant (**) when the *p*-value was less than 0.01.

## 5. Conclusions

The present study explores the potential of a relatively lesser-known fruit for its neuroprotective effects, expanding knowledge in the field of natural therapies for neurodegenerative diseases. The results from both direct cell viability tests and models of oxidative stress related to PD and AD highlight the plant’s potential therapeutic value. MAO-A and MAO-B inhibition provide mechanistic insights into how the juice confers neuroprotection. Additionally, UHPLC-HRMS analysis offers a robust analytical characterization of the bioactive components responsible for the observed effects.

Due to its capacity to stimulate viability on neurons under toxic conditions and slow down the degradation of monoaminergic neurotransmitters through the inhibition of MAO-A and MAO-B, *C. album* berry juice has shown high potential in the prevention and treatment of neurodegenerative diseases, such as PD and AD. This ability, together with its capacity to scavenge free radicals and reduce ROS production, indicates a neuroprotective interest in this juice and supports further research aimed at elucidating the mechanism of action of the phytochemical components of the juice.

## Figures and Tables

**Table 1:** High-resolution mass spectrometry (HRMS) and HRMS/MS spectra, in negative ionization mode of the phenolic compounds identified in the juice of *Corema album* berries.

Phenolic Compounds	Area	RT (min)	Molecular Formula	[M − H]^−^ ^m/z Calculated Value^	[M − H]^−^ ^m/z Experim. Value^	Accurate Mass Error (ppm)	Fragment Ions (m/z)	Assignment	Accurate Mass Error (ppm)
**(−)-Epigallocatechin**	7.99 × 10^6^	3.51	C_15_H_14_O_7_	305.06668	305.06644	−0.80	109.02952 125.02431 137.0244 167.03503 219.0665	[M − H -C_9_H_9_O_5_]^−^ [M − H -C_9_H_9_O_4_]^−^ [M − H -C_8_H_9_O_4_]^−^ [M − H -C_7_H_7_O_3_]^−^ [M − H -C_3_H_4_O_3_]^−^	0.16626 −0.84079 −0.15469 0.32436 1.3425
**5-O-Caffeoilquinic**	2.00 × 10^9^	3.86	C_16_H_18_O_9_	353.08781	353.08765	−0.46	59.01387 85.02955 93.03458 127.04009 191.0563	[M − H -C_10_H_13_O_8_]^−^ [M − H -C_9_H_7_O_3_]^−^	0.4076 0.5721 −0.064 0.17686 1.02028
**6,7-dihydroxycoumarin** **(Esculetin)**	5.26 × 10^6^	3.94	C_9_H_6_O_4_	177.01933	177.01923	−0.59			
**Caffeic acid**	2.70 × 10^7^	4.04	C_9_H_8_O_4_	179.03498	179.03494	−0.21	89.03959 107.05027 134.03738 135.04523	[M − H -C_2_O_3_]^−^ [M − H -COO]^−^	−0.91051 0.28099 0.40312 0.57056
**(−)-Epicatechin**	2.73 × 10^7^	4.35	C_15_H_14_O_6_	289.07176	289.07172	−0.15	245.08188 203.07158 137.02446 123.04521 109.0295	[M − H -COO]^−^ [M − H -C_3_H_2_O_3_]^−^ [M − H -C_8_H_6_O_4_]^−^ [M − H -C_9_H_8_O_4_]^−^	−0.20947 1.02909 0.29074 0.50219 −0.04366
**Phloretic acid**	4.74 × 10^6^	4.77	C_9_H_10_O_3_	165.05572	165.05556	−0.99			
**Myricetin-3-O-galactoside**	5.00 × 10^7^	5.14	C_21_H_20_O_13_	479.08311	479.08286	−0.53	271.02509 287.01993 316.02249	[M − H -C_6_H_10_O_5_]^−^	1.01632 0.68977 0.06789
**Quercetin-3-O-galactoside (Hiperoside)**	2.36 × 10^8^	5.57	C_21_H_20_O_12_	463.0882	463.08844	0.52	227.03499 243.02982 255.02994 271.02502 300.02765	[M − H -C_5_H_10_O_3_]^−^ [M − H -C_6_H_10_O_5_]^−^	0.03701 −0.34698 0.148 0.79112 0.32972
**Ellagic acid**	1.30 × 10^6^	5.69	C_14_H_6_O_8_	300.99899	300.9986	−1.31			
**Naringin**	4.78 × 10^6^	5.79	C_27_H_32_O_14_	579.17193	579.17133	−1.04			
**Kaempferol-3-O-glucoside**	3.15 × 10^7^	6.08	C_21_H_20_O_11_	447.09328	447.09308	−0.45	183.04517 211.04045 227.03519 255.03006 284.03284	[M − H -C_5_H_10_O_3_]^−^ [M − H -C_6_H_10_O_5_]^−^	0.0875 1.80558 0.91073 0.62665 0.69328
**Pinocembrin**	1.24 × 10^8^	8.08	C_15_H_12_O_4_	255.06628	255.06624	−0.16	65.00332 83.0139 107.01388 151.00374 171.04523		0.44285 0.61143 0.26042 0.38677 0.45047

**Table 2:** In vitro scavenging activity of *Corema album* berry juice and gallic acid. IC_50_ calculated from *n* = 3 samples expressed as the mean ± SE in µg/mL.

	Juice	Gallic Acid
DPPH	412.7 µg/mL ± 4.4	3.72 µg/mL ± 0.04
ABTS	112.4 µg/mL ± 9.6	28.6 µg/mL ± 0.1
Hydroxyl	221.2 µg/mL ± 2.6	331.4 µg/mL ± 2.1
Superoxide	426.9 µg/mL ± 0.2	12.4 µg/mL ± 0.02

**Table 3:** In vitro inhibitory effect of *Corema album* berry juice and clorgyline/selegiline on the activity of MAO-A and MAO-B, respectively. IC_50_ calculated from *n* = 3 samples expressed as the mean ± SE in µg/mL.

	Juice	Clorgyline	Selegiline
MAO-A	87.21 µg/m ± 0.01	5.04 ng/mL ± 0.33	-
MAO-B	56.50 µg/mL ± 0.006	-	49.55 ng/mL ± 8.52

## Data Availability

The original contributions presented in the study are included in the article, further inquiries can be directed to the corresponding author.
